# (*E*)-1,2-Diphenyl­vinyl *p*-toluene­sulfonate

**DOI:** 10.1107/S160053680802607X

**Published:** 2008-08-20

**Authors:** Dongmei Cui, Qian Meng, Chen Zhang, Jianming Gu

**Affiliations:** a Zhejiang University of Technology, College of Pharmaceutical Science, Hangzhou 310014, People’s Republic of China; b Zhejiang University, Hangzhou 310058, People’s Republic of China

## Abstract

The title compound, C_21_H_18_O_3_S, is the *E* isomer, the ester ­oxy link being *trans* to one of the phenyl groups. The planes of the phenyl substituents at the vinyl C atoms form a dihedral angle of 66.32 (7)° with each other. The vinyl group shows noticeable non-planarity, the C(Ph)—C=C—C(Ph) torsion angle being 8.4 (3)°.

## Related literature

For related literature, see: Ishikawa *et al.* (2001 ▶); Peterson & Indelicato (1968 ▶); Yoshihiro & Atsushi (1993 ▶); Larson (1970 ▶).
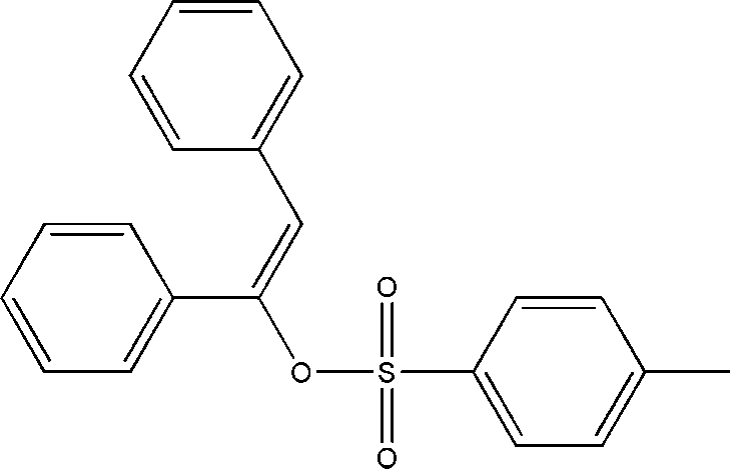


## Experimental

### 

#### Crystal data

C_21_H_18_O_3_S
*M* *_r_* = 350.43
Monoclinic, 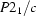

*a* = 19.8000 (7) Å
*b* = 5.8289 (2) Å
*c* = 15.5228 (7) Å
β = 97.2226 (12)°
*V* = 1777.31 (12) Å^3^
*Z* = 4
Mo *K*α radiation
μ = 0.20 mm^−1^
*T* = 296 (1) K
0.50 × 0.50 × 0.20 mm

#### Data collection

Rigaku R-AXIS RAPID diffractometer
Absorption correction: multi-scan (*ABSCOR*; Higashi, 1995 ▶) *T*_min_ = 0.879, *T*_max_ = 0.961
16497 measured reflections
4064 independent reflections
2454 reflections with *I* > 2σ(*I*)
*R*_int_ = 0.037

#### Refinement

*R*[*F* ^2^ > 2σ(*F* ^2^)] = 0.035
*wR*(*F* ^2^) = 0.115
*S* = 1.01
4064 reflections
245 parameters
H-atom parameters constrained
Δρ_max_ = 0.32 e Å^−3^
Δρ_min_ = −0.37 e Å^−3^

### 

Data collection: *PROCESS-AUTO* (Rigaku, 1998 ▶); cell refinement: *PROCESS-AUTO*; data reduction: *CrystalStructure* (Rigaku/MSC, 2004 ▶); program(s) used to solve structure: *SIR97* (Altomare *et al.*, 1999 ▶); program(s) used to refine structure: *CRYSTALS* (Betteridge *et al.*, 2003); molecular graphics: *ORTEP-3 for Windows* (Farrugia, 1997 ▶); software used to prepare material for publication: *CrystalStructure*.

## Supplementary Material

Crystal structure: contains datablocks global, I. DOI: 10.1107/S160053680802607X/ya2077sup1.cif

Structure factors: contains datablocks I. DOI: 10.1107/S160053680802607X/ya2077Isup2.hkl

Additional supplementary materials:  crystallographic information; 3D view; checkCIF report

## Figures and Tables

**Fig. 1.**
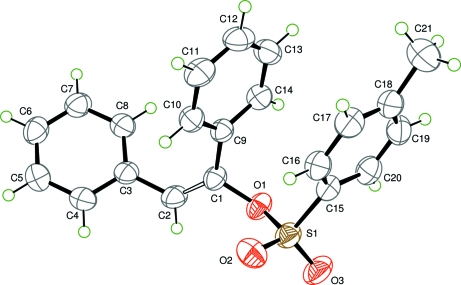
Molecular structure of the title compound, showing 30% probability displacement ellipsoids and atom numbering scheme.

## supplementary crystallographic information

### Comment

The enol sulfonates have been extensively studied as substrates in solvolytic displacement reactions (Peterson & Indelicato, 1968) and as starting materials or intermediates in the synthesis of various medicines and agrichemicals (Yoshihiro & Atsushi, 1993). They also play important role in the synthesis of cephalosporin derivates (Ishikawa *et al.*, 2001). As a part of our studies of enol sulfonates, we synthesized the title compound and determined its crystal structure.

The title compound is shown to be an E-isomer with the ester O1 oxygen in *trans*-position to the phenyl carbon atom C3. The C1=C2 double bond environment is notceably non-planar with the torsion angle C3—C2—C1—C9 being equal to 8.4 (3)°. The planes of Ph-groups C3—C8 and C9—C14 form dihedral angle of 66.32 (7)° with each other, whereas the C9—C14 plane is approximately parallel to the benzene plane C15—C20 of *p*-toluenesulfonic group; corresponding dihedral angle is equal to 10.5 (2)°.

### Experimental

1,2-Diphenylethanone (0.84 mg, 4.3 mmol) in 2 ml of THF was slowly added to 5.00 mmol of freshly prepared lithium diisopropylamide at 195 K and stirred for 1 h. The resulting pale yellow solution was transferred to a solution of 3.0 g (9.2 mmol) of toluenesulfonic anhydride in 20 ml of THF at 273 K *via* a double-tip needle. This mixture was warmed to room temperature over a 3 h period and then poured into 200 ml of cold saturated NaHCO_3_. Extraction into ether (250 ml) was followed by washing with brine (250 ml) and water (250 ml) and drying (MgSO_4_). Evaporation of the solvent left a yellow-brown oil (0.94 g, 80% yield). Recrystallization from 20% ether/pentane gave colourless crystals.

### Refinement

All H atoms were treated as riding atoms at distances of 0.93 (benzene), 0.96 (methyl) and 0.93 Å (methylene), with *U*_iso_(H) = 1.2*U*_eq_ of the carrying atom.

### Crystal data

**Table:**

C_21_H_18_O_3_S	*F*(000) = 736.00
*M**_r_* = 350.43	*D*_x_ = 1.310 Mg m^−^^3^
Monoclinic, *P*2_1_/*c*	Mo *K*α radiation, λ = 0.71075 Å
Hall symbol: -P 2ybc	Cell parameters from 11320 reflections
*a* = 19.8000 (7) Å	θ = 3.1–27.4°
*b* = 5.8289 (2) Å	µ = 0.20 mm^−^^1^
*c* = 15.5228 (7) Å	*T* = 296 K
β = 97.2226 (12)°	Plate, colourless
*V* = 1777.31 (12) Å^3^	0.50 × 0.50 × 0.20 mm
*Z* = 4	

### Data collection

**Table:**

Rigaku R-AXIS RAPID diffractometer	2454 reflections with *I* > 2σ(*I*)
Detector resolution: 10.00 pixels mm^-1^	*R*_int_ = 0.037
ω scans	θ_max_ = 27.5°
Absorption correction: multi-scan (*ABSCOR*; Higashi, 1995)	*h* = −25→25
*T*_min_ = 0.879, *T*_max_ = 0.961	*k* = −6→7
16497 measured reflections	*l* = −20→20
4064 independent reflections	

### Refinement

**Table:**

Refinement on *F*^2^	H-atom parameters constrained
*R*[*F*^2^ > 2σ(*F*^2^)] = 0.035	*w* = 1/[0.0007*F*_o_^2^ + σ(*F*_o_^2^)]/(4*F*_o_^2^)
*wR*(*F*^2^) = 0.115	(Δ/σ)_max_ < 0.001
*S* = 1.01	Δρ_max_ = 0.32 e Å^−^^3^
4064 reflections	Δρ_min_ = −0.37 e Å^−^^3^
245 parameters	Extinction correction: Larson (1970)
0 restraints	Extinction coefficient: 704 (41)

### Special details

**Table:**

Geometry. ENTER SPECIAL DETAILS OF THE MOLECULAR GEOMETRY
Refinement. Refinement using all reflections. The weighted *R*-factor (*wR*) and goodness of fit (*S*) are based on *F*^2^. *R*-factor (gt) are based on *F*. The threshold expression of *F*^2^ > 2.0 σ(*F*^2^) is used only for calculating *R*-factor (gt).

### Fractional atomic coordinates and isotropic or equivalent isotropic displacement parameters (Å^2^)

**Table:**

	*x*	*y*	*z*	*U*_iso_*/*U*_eq_	
S1	0.18392 (2)	0.36995 (9)	0.40582 (3)	0.04917 (14)	
O1	0.24688 (6)	0.5425 (2)	0.41891 (8)	0.0490 (3)	
O2	0.20815 (6)	0.1464 (2)	0.39044 (10)	0.0626 (4)	
O3	0.14984 (6)	0.4139 (2)	0.47932 (9)	0.0692 (4)	
C1	0.30475 (8)	0.5083 (2)	0.37345 (12)	0.0423 (4)	
C2	0.36167 (9)	0.4467 (3)	0.42212 (12)	0.0504 (5)	
C3	0.43026 (8)	0.4338 (3)	0.39505 (12)	0.0457 (5)	
C4	0.47282 (10)	0.2516 (3)	0.42163 (13)	0.0588 (6)	
C5	0.53825 (10)	0.2424 (3)	0.40017 (14)	0.0636 (6)	
C6	0.56265 (9)	0.4158 (3)	0.35334 (13)	0.0564 (6)	
C7	0.52180 (9)	0.5978 (3)	0.32689 (13)	0.0564 (6)	
C8	0.45589 (9)	0.6074 (3)	0.34753 (12)	0.0528 (5)	
C9	0.29281 (8)	0.5511 (2)	0.27937 (12)	0.0399 (4)	
C10	0.31048 (9)	0.3861 (3)	0.22233 (12)	0.0463 (5)	
C11	0.29633 (10)	0.4197 (3)	0.13398 (12)	0.0563 (6)	
C12	0.26427 (10)	0.6160 (3)	0.10150 (12)	0.0570 (6)	
C13	0.24713 (9)	0.7817 (3)	0.15732 (13)	0.0554 (6)	
C14	0.26083 (8)	0.7515 (3)	0.24614 (12)	0.0469 (5)	
C15	0.13442 (8)	0.4713 (3)	0.31215 (12)	0.0437 (5)	
C16	0.13277 (9)	0.3540 (3)	0.23505 (12)	0.0490 (5)	
C17	0.09787 (9)	0.4460 (3)	0.16026 (13)	0.0551 (5)	
C18	0.06416 (9)	0.6543 (3)	0.16162 (13)	0.0558 (6)	
C19	0.06504 (9)	0.7662 (3)	0.24064 (16)	0.0588 (6)	
C20	0.09969 (9)	0.6786 (3)	0.31559 (13)	0.0538 (5)	
C21	0.02903 (12)	0.7611 (4)	0.07986 (17)	0.0876 (9)	
H2	0.3579	0.4074	0.4794	0.060*	
H4	0.4570	0.1340	0.4543	0.071*	
H5	0.5659	0.1178	0.4176	0.076*	
H6	0.6070	0.4097	0.3395	0.068*	
H7	0.5383	0.7155	0.2949	0.068*	
H8	0.4284	0.7320	0.3293	0.063*	
H10	0.3320	0.2521	0.2436	0.056*	
H11	0.3086	0.3082	0.0960	0.068*	
H12	0.2543	0.6363	0.0418	0.068*	
H13	0.2261	0.9158	0.1353	0.066*	
H14	0.2488	0.8644	0.2837	0.056*	
H16	0.1550	0.2138	0.2332	0.059*	
H17	0.0970	0.3668	0.1081	0.066*	
H19	0.0416	0.9038	0.2429	0.071*	
H20	0.1000	0.7564	0.3679	0.065*	
H211	0.0549	0.7310	0.0327	0.105*	
H212	0.0255	0.9238	0.0878	0.105*	
H213	−0.0157	0.6967	0.0668	0.105*	

### Atomic displacement parameters (Å^2^)

**Table:**

	*U*^11^	*U*^22^	*U*^33^	*U*^12^	*U*^13^	*U*^23^
S1	0.0464 (2)	0.0604 (3)	0.0419 (3)	−0.0014 (2)	0.0103 (2)	0.0001 (2)
O1	0.0444 (6)	0.0644 (8)	0.0398 (7)	−0.0042 (5)	0.0116 (5)	−0.0115 (6)
O2	0.0705 (8)	0.0510 (8)	0.0651 (10)	0.0076 (6)	0.0040 (7)	0.0045 (7)
O3	0.0573 (7)	0.1060 (12)	0.0483 (9)	−0.0027 (7)	0.0228 (6)	0.0018 (8)
C1	0.0413 (8)	0.0491 (10)	0.0376 (10)	−0.0001 (7)	0.0088 (7)	−0.0053 (8)
C2	0.0480 (9)	0.0689 (12)	0.0343 (10)	−0.0031 (9)	0.0054 (8)	0.0055 (9)
C3	0.0437 (9)	0.0600 (11)	0.0325 (10)	−0.0006 (8)	0.0018 (7)	0.0033 (8)
C4	0.0571 (11)	0.0653 (13)	0.0539 (13)	0.0045 (10)	0.0070 (9)	0.0190 (10)
C5	0.0534 (11)	0.0721 (14)	0.0648 (14)	0.0137 (10)	0.0058 (10)	0.0122 (12)
C6	0.0426 (9)	0.0760 (14)	0.0501 (12)	−0.0005 (10)	0.0040 (8)	−0.0036 (11)
C7	0.0472 (9)	0.0674 (13)	0.0548 (13)	−0.0070 (9)	0.0073 (9)	0.0082 (10)
C8	0.0494 (10)	0.0567 (11)	0.0516 (12)	0.0012 (9)	0.0042 (8)	0.0091 (10)
C9	0.0379 (8)	0.0456 (9)	0.0362 (10)	−0.0007 (7)	0.0053 (7)	−0.0021 (8)
C10	0.0493 (9)	0.0477 (10)	0.0425 (11)	0.0045 (8)	0.0090 (8)	−0.0040 (9)
C11	0.0644 (11)	0.0656 (13)	0.0404 (12)	−0.0031 (10)	0.0130 (9)	−0.0133 (10)
C12	0.0605 (11)	0.0732 (14)	0.0367 (11)	−0.0102 (10)	0.0032 (9)	0.0061 (10)
C13	0.0543 (10)	0.0537 (12)	0.0568 (13)	−0.0014 (9)	0.0020 (9)	0.0139 (10)
C14	0.0489 (9)	0.0448 (10)	0.0475 (12)	−0.0012 (8)	0.0082 (8)	−0.0029 (9)
C15	0.0386 (8)	0.0485 (10)	0.0450 (11)	−0.0022 (7)	0.0093 (7)	−0.0020 (8)
C16	0.0466 (9)	0.0489 (10)	0.0516 (12)	−0.0001 (8)	0.0073 (8)	−0.0067 (9)
C17	0.0523 (10)	0.0654 (12)	0.0471 (12)	−0.0056 (10)	0.0044 (9)	−0.0079 (10)
C18	0.0415 (9)	0.0679 (13)	0.0576 (14)	−0.0036 (9)	0.0046 (9)	0.0085 (11)
C19	0.0461 (10)	0.0553 (12)	0.0763 (16)	0.0075 (9)	0.0124 (10)	0.0068 (11)
C20	0.0513 (10)	0.0544 (11)	0.0580 (13)	0.0020 (9)	0.0160 (9)	−0.0078 (10)
C21	0.0740 (14)	0.105 (2)	0.0801 (19)	0.0060 (14)	−0.0029 (13)	0.0261 (16)

### Geometric parameters (Å, °)

**Table:**

S1—O1	1.5946 (12)	C17—C18	1.387 (2)
S1—O2	1.4189 (14)	C18—C19	1.387 (3)
S1—O3	1.4200 (15)	C18—C21	1.503 (3)
S1—C15	1.7508 (17)	C19—C20	1.373 (2)
O1—C1	1.433 (2)	C2—H2	0.930
C1—C2	1.325 (2)	C4—H4	0.930
C1—C9	1.471 (2)	C5—H5	0.930
C2—C3	1.473 (2)	C6—H6	0.930
C3—C4	1.386 (2)	C7—H7	0.930
C3—C8	1.386 (2)	C8—H8	0.930
C4—C5	1.379 (2)	C10—H10	0.930
C5—C6	1.368 (3)	C11—H11	0.930
C6—C7	1.366 (2)	C12—H12	0.930
C7—C8	1.383 (2)	C13—H13	0.930
C9—C10	1.382 (2)	C14—H14	0.930
C9—C14	1.396 (2)	C16—H16	0.930
C10—C11	1.379 (2)	C17—H17	0.930
C11—C12	1.374 (2)	C19—H19	0.930
C12—C13	1.368 (3)	C20—H20	0.930
C13—C14	1.383 (2)	C21—H211	0.960
C15—C16	1.375 (2)	C21—H212	0.960
C15—C20	1.395 (2)	C21—H213	0.960
C16—C17	1.382 (2)		
			
O1—S1—O2	108.97 (7)	C1—C2—H2	116.6
O1—S1—O3	103.10 (8)	C3—C2—H2	116.6
O1—S1—C15	103.89 (7)	C3—C4—H4	119.6
O2—S1—O3	120.38 (9)	C5—C4—H4	119.6
O2—S1—C15	109.63 (8)	C4—C5—H5	119.8
O3—S1—C15	109.48 (8)	C6—C5—H5	119.8
S1—O1—C1	120.67 (10)	C5—C6—H6	120.1
O1—C1—C2	115.62 (16)	C7—C6—H6	120.1
O1—C1—C9	115.25 (13)	C6—C7—H7	119.9
C2—C1—C9	129.13 (17)	C8—C7—H7	119.9
C1—C2—C3	126.85 (18)	C3—C8—H8	119.6
C2—C3—C4	120.13 (17)	C7—C8—H8	119.6
C2—C3—C8	121.81 (16)	C9—C10—H10	119.9
C4—C3—C8	117.94 (16)	C11—C10—H10	119.9
C3—C4—C5	120.82 (19)	C10—C11—H11	119.7
C4—C5—C6	120.35 (19)	C12—C11—H11	119.7
C5—C6—C7	119.88 (18)	C11—C12—H12	120.1
C6—C7—C8	120.15 (19)	C13—C12—H12	120.1
C3—C8—C7	120.86 (17)	C12—C13—H13	119.7
C1—C9—C10	119.73 (15)	C14—C13—H13	119.7
C1—C9—C14	121.18 (16)	C9—C14—H14	120.1
C10—C9—C14	119.02 (17)	C13—C14—H14	120.1
C9—C10—C11	120.18 (17)	C15—C16—H16	120.2
C10—C11—C12	120.66 (19)	C17—C16—H16	120.2
C11—C12—C13	119.71 (18)	C16—C17—H17	119.4
C12—C13—C14	120.55 (18)	C18—C17—H17	119.4
C9—C14—C13	119.88 (17)	C18—C19—H19	119.2
S1—C15—C16	120.30 (13)	C20—C19—H19	119.2
S1—C15—C20	119.19 (14)	C15—C20—H20	120.5
C16—C15—C20	120.42 (16)	C19—C20—H20	120.5
C15—C16—C17	119.51 (17)	C18—C21—H211	109.5
C16—C17—C18	121.17 (19)	C18—C21—H212	109.5
C17—C18—C19	118.19 (18)	C18—C21—H213	109.5
C17—C18—C21	121.5 (2)	H211—C21—H212	109.5
C19—C18—C21	120.32 (19)	H211—C21—H213	109.5
C18—C19—C20	121.60 (18)	H212—C21—H213	109.5
C15—C20—C19	119.09 (19)		
			
O2—S1—O1—C1	32.69 (14)	C3—C4—C5—C6	1.1 (3)
O3—S1—O1—C1	161.67 (12)	C4—C5—C6—C7	−0.7 (3)
O1—S1—C15—C16	106.36 (15)	C5—C6—C7—C8	0.1 (2)
O1—S1—C15—C20	−70.21 (15)	C6—C7—C8—C3	−0.0 (2)
C15—S1—O1—C1	−84.11 (13)	C1—C9—C10—C11	176.68 (16)
O2—S1—C15—C16	−9.98 (17)	C1—C9—C14—C13	−176.66 (15)
O2—S1—C15—C20	173.46 (14)	C10—C9—C14—C13	0.2 (2)
O3—S1—C15—C16	−144.06 (15)	C14—C9—C10—C11	−0.3 (2)
O3—S1—C15—C20	39.38 (17)	C9—C10—C11—C12	−0.4 (2)
S1—O1—C1—C2	−110.89 (15)	C10—C11—C12—C13	1.0 (3)
S1—O1—C1—C9	70.28 (17)	C11—C12—C13—C14	−1.0 (2)
O1—C1—C2—C3	−170.22 (16)	C12—C13—C14—C9	0.4 (2)
O1—C1—C9—C10	−127.99 (16)	S1—C15—C16—C17	−174.80 (14)
O1—C1—C9—C14	48.9 (2)	S1—C15—C20—C19	175.21 (14)
C2—C1—C9—C10	53.4 (2)	C16—C15—C20—C19	−1.4 (2)
C2—C1—C9—C14	−129.8 (2)	C20—C15—C16—C17	1.7 (2)
C9—C1—C2—C3	8.4 (3)	C15—C16—C17—C18	−0.3 (2)
C1—C2—C3—C4	−138.5 (2)	C16—C17—C18—C19	−1.4 (2)
C1—C2—C3—C8	45.6 (2)	C16—C17—C18—C21	176.93 (19)
C2—C3—C4—C5	−177.00 (18)	C17—C18—C19—C20	1.8 (2)
C2—C3—C8—C7	176.40 (18)	C21—C18—C19—C20	−176.57 (19)
C4—C3—C8—C7	0.4 (2)	C18—C19—C20—C15	−0.4 (2)
C8—C3—C4—C5	−1.0 (2)		
